# Effect of insulin-like growth factor 1 gene on growth traits of Kejobong goat and its growth analysis

**DOI:** 10.14202/vetworld.2020.127-133

**Published:** 2020-01-18

**Authors:** Dela Ayu Lestari, Takuro Oikawa, Sutopo Sutopo, Endang Purbowati, Asep Setiaji, Edy Kurnianto

**Affiliations:** 1 Department of Animal Science, Faculty of Animal and Agricultural Sciences, Diponegoro University, Tembalang Campus, Semarang, Central Java 50275, Indonesia; 2 Department of Subtropical Agro-Environmental Sciences, Faculty of Agriculture, University of the Ryukyus, Nishihara, Okinawa 903-0213, Japan; 3 United Graduate School of Agricultural Sciences, Kagoshima University, Korimoto, Kagoshima 890-8580, Japan

**Keywords:** genetic markers, goat, growth analysis, growth traits, insulin-like growth factor 1

## Abstract

**Aim:**

This study aimed to identify the effect of the insulin-like growth factor 1 (*IGF1*) gene on growth, to uncover the genetic marker at the *IGF1* gene, and to predict growth performance by analyzing growth models of Kejobong goats based on their genotype.

**Materials and Methods:**

DNA and records of body weight (BW) and body measurements (BM) of Kejobong goats were collected, the *IGF1* gene was amplified from the DNA template by polymerase chain reaction (PCR); the PCR products were then sequenced to determine single nucleotide polymorphisms (SNP). Linear mixed model (LMM) was used to analyze the association between SNP and growth traits. Four non-linear growth models were analyzed using non-LMM to describe the growth model and to compare the growth within genotypes.

**Results:**

An SNP at intron 4 (g5752G→C) genotyped into GG and CC was significantly associated with BW and BM. Goats of genotype GG had a significantly higher BW and BM (p<0.05) than those of genotype CC. Growth analysis showed that the von Bertalanffy model was the most fit for describing BW, the Brody model for chest width and hip height, the Gompertz and Logistic models for heart girth, and the von Bertalanffy and Gompertz models for hip width.

**Conclusion:**

An SNP at intron 4 of the *IGF1* gene was associated with the growth trait and was usable as a genetic marker candidate for improvement of growth traits of Kejobong goats while von Bertalanffy model provides proper and accurate estimates of parameters to describe the growth performance of Kejobong goats.

## Introduction

Growth traits have always attracted much interest in the production of meat animals. In Indonesia, most farmers have maintained traditional livestock farming systems and have depended on local livestock for their main source of income. The Kejobong goat is known as an indigenous Indonesian breed raised by a semi-intensive animal farming system by local farmers. This goat has been confirmed to be the progeny of a cross between Kacang and Etawah grade goats [1,2]. The Kejobong goat is known not only for its prolific traits but also for its high rate of growth, good carcass composition, and good reproductive performance [3]. Nonetheless, the genetic improvement of Kejobong goats has been slow because only a few genetic studies have been done on its growth traits.

One of the main goals with meat animals is identifying those with superior growth performance and using them in a cyclical system of animal breeding. Due to a lack of animal pedigree and production records, it is difficult to improve the performance of local breeds over a short period of time by traditional breeding programs. Recently, therefore, major breeders have focused on using DNA markers for improving breeds through marker-assisted selection (MAS) and/or marker-assisted introgression. The first step in this approach is to identify genes that determine some markers of growth performance. Growth performance is the most common trait used for evaluating the economic value of animals. Physiologically, growth is the effect of a complex process that regulates ­neuroendocrine pathways, among which the somatotrophic axis (growth hormone/insulin-like growth factor 1 [*IGF1*] axis) plays a substantial part in postnatal growth and metabolism in mammals [4]. *IGF1*, one of the ­somatotrophic axis components, encourages cell proliferation, skeletal growth, and protein synthesis as anabolic processes [5]. The *IGF1* gene sequence in goats has been determined to be 6,784 bp long (D26119), located on chromosome 5 and comprising three leader exons (1w, 1, and 2) and three exons (3, 4, and 6) [6]. Consequently, the *IGF1* gene is expected to be one of the candidate gene markers associated with growth traits.

The growth of animals is evaluated by aspects such as weight at maturity, growth rate, and growth acceleration, which can be illustrated with the growth model. The growth model can also describe and express the animal’s maximal genetic potential under existing environmental conditions [7]. Moreover, modeling the growth of animals can quantify the animal’s optimal growth and determine the right slaughtering time. Thus, analysis of the growth model provides worthwhile information for designing selection programs and for planning farm management strategies and decision-making on genetic selection by predicting future growth at any age [7,8].

This study aimed to identify the effect of the *IGF1* gene on growth, to uncover the genetic marker at the *IGF1* gene, and to predict growth performance by analyzing growth models of Kejobong goats.

## Materials and Methods

### Ethical approval

The protocol was based on the standard rule of animal treating as appointed in the Republic of Indonesia’s law, that is, number 41, 2014.

### Sample collection and phenotypic data

A total of 35 blood samples and phenotypic data on the Kejobong goat were collected from Purbalingga District, Central Java Province, Indonesia. Samples were taken from 10 bucks and 25 does. The sampling and research locations were based on purposive sampling methods and selected based on the density of the Kejobong goat population. The animals were raised under semi-intensive management and traditional farming procedures by four local livestock-farming groups.

Body weight (BW) of the goats was taken with a hanging scale. Chest width (CW), hip height (HH), and hip width (HW) were measured with a measuring stick and heart girth (HG) with a measuring tape. BW and body measurements(BM) were taken between ages 0-15 days, 16-31 days, 32-47 days, 48-63 days, 64-79 days, 80-95 days, 96-111 days, and 112-127 days. Blood samples for DNA analysis drawn from the jugular venous with a 3 cc spuit and collected in Vacutainer blood collection tubes with an anticoagulant (EDTA).

### DNA extraction, polymerase chain reaction (PCR), and sequencing

DNA was extracted with a gSYNC DNA Mini Kit (Geneaid Biotech Ltd.), according to the manufacturer’s standard protocol. *IGF1* exon 4 was amplified using forward primer 5’-gctgggtgtagcagtgaaca -3’ and reverse primer 5’- gttgcttcagccgcataact -3’ [9]. PCR was carried out in a total volume of 50 µL comprising 25 µL KAPA2G Fast ReadyMix + Dye (Kapa Biosystems Ltd.), 1 µL forward primer and 1 µL reverse primer (Integrated DNA Technologies Pte. Ltd.), 20 µL double-distilled water, and 3 µL DNA template. Amplification (PCR) was carried out with the following conditions: Pre-denaturation (at 94°C for 5 min); 35 cycles of denaturation (at 94°C for 30 s), primer annealing (at 56°C for 30 s), elongation (at 72°C for 30 s), and post-elongation (at 72°C for 10 min). PCR products were then electrophoresed with 1% agarose gel at 100 V for 20 min and visualized under ultraviolet transilluminator. The amplicon was then purified and sequenced through the 1^st^ Base DNA Sequencing Services, Singapore.

## Statistical analysis

Allele frequencies were estimated by the gene-counting method, as follows:

p^2^+2pq+q^2^ = 1,

Where p is allele frequency of the first allele and q allele frequency of the second allele.

Genotype distribution was tested for determining Hardy-Weinberg Equilibrium (HWE) by Chi-square analysis, as follows:


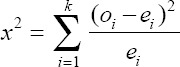


Where *χ^2^* is the Chi-square value; *o_i_* the observed value of genotype frequency, e_i_ the expected value of genotype frequency, *χ^2^* the table using 5% significance level for the HWE test.

Heterozygosity (H) was estimated, as follows:


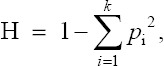


Where H is the value of heterozygosity and *p*_i_ the frequency of the *i*^th^ of *k* alleles.

The *IGF1* gene sequence was analyzed with the use of molecular evolutionary genetics analysis version 6.0 [10] to uncover polymorphisms in the animals. Clustal W was used to align the sequence [11]. The *IGF1* gene sequence of Kejobong goats was also aligned with the *Capra hircus* *IGF1* gene sequence (D26119) from GenBank [6].

The association between single nucleotide polymorphisms (SNP) and BW/BM was analyzed with the use of the linear mixed model (LMM) of statistical analysis system (SAS) version 9.3 [12]. The model used was

y_ijkl_ = µ+G_i_+F_j_+u_k_+b_1_ɑ_ijkl_+b_2_ɑ^2^_ijkl_+e_ijkl_

Where y_ijkl_ is the observed value of a dependent variable (BW/BM); µ the overall mean of the population; G_i_ the fixed effect of i^th^ genotype (i = 1 [GG], 2 [GC], 3 [CC]); F_j_ the fixed effect of j^th^ farm group (j = 1, 2, 3, 4); u_k_ the random effect of k^th^ individual; b_1_ and b_2_ the linear and quadratic coefficients of partial regression, respectively; ɑ_ijkl_ age in days of a covariate and e_ijkl_ the random residual for Y_ijkl_. The difference in the least-square means of the genotypes was determined by the Tukey-Kramer test [13].

In this study, the following four non-linear models used for describing animal growth models were compared: Brody [14], von Bertalanffy [15], Logistic [16], and Gompertz [17] (Table-1).

**Table-1:** Growth models for constructing a growth model.

Model	Function*
Brody	y=A* (1–B exp^-C*age^)
Von Bertalanffy	y=A* (1–B exp^-C*age^)^3^
Logistic	y=A/(1+B exp^-C*age^)
Gompertz	y=A*exp (–B exp^-C*age^)

* y=Observed body weight/body measurements, A=The estimated of mature body weight/body measurements, B=The integration constant, C=The growth rate constant, Age, the animal age in day and exp, Napier’s constant the base of the natural logarithm (2.7183)

To obtain growth model parameters, non-LMM (NLMM) analysis was performed with SAS version 9.3 [12] for estimating parameters of fixed and random effects. BW/BM as dependent variables are influenced by quantitative (age) and qualitative (group farm/type of birth and genotype) variables. Therefore, dummy variables have been created to assess the effect of qualitative variables on dependent variables and regression [18].

Under the assumption of normality of random residuals, alternative models were evaluated by the −2 log-likelihood, Akaike information criterion (AIC) [19], Bayesian information criterion (BIC) [20], and the residual variances (σ^2^_e_). AIC and BIC were calculated, as follows:


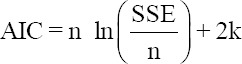



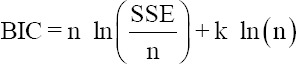


Where n is the number of observations; SSE the sum square errors, and k the number of parameters. Smaller values of AIC, BIC, or σ^2^_e_ indicate the best fit of the model to the data.

## Results

**Figure-1:**
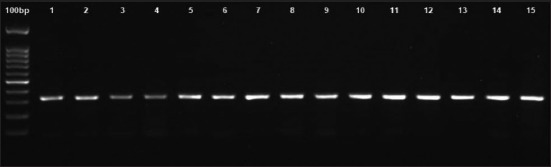
Polymerase chain reaction result.

**Figure-2:**
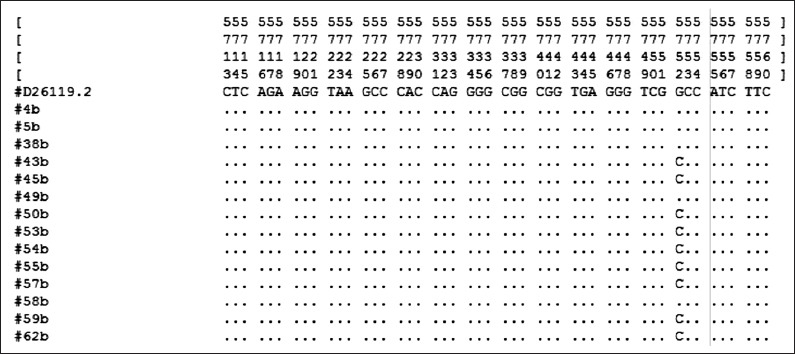
Alignment result.

**Figure-3:**
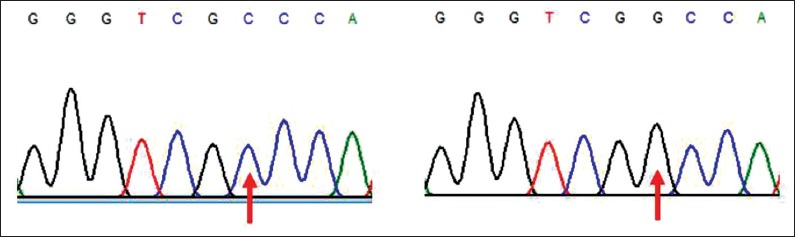
Identified single nucleotide polymorphisms.

PCR showed that the *IGF1* gene was well amplified. The amplification generates about 322 bp sequences (Figure-1). After alignment and blast checking, the sequences comprised 71 bp of partial intron 3, 182 bp of exon 4, and 69 bp of partial intron 4. The SNP was observed in the animals at intron 4 as a transversion mutation. Likewise, when the sequence was aligned with D26119 [6], SNP was identified at the same location (Figure-2) g5752G→C, a parsimonious form designated here as GG and CC genotypes (Figure-3). The estimated allele and genotype frequency of the *IGF1* gene in Kejobong goats were 43% and 57% for G and C, respectively. The frequency of genotype GG and CC was 43% and 57%, respectively, while that of genotype GC was not observed in this study. The genotype distribution of Kejobong goats was statistically different (p<0.05) from HWE, and the frequency of heterozygosity was 49% (Table-2).

**Table-2:** Estimated allele and genotype frequency.

Variable measured	Genotype			Allele		H	χ^2^
	
	GG	GC	CC	G	C		
Frequencies	0.43	0.00	0.57	0.43	0.57	0.49	35.00*
Observation	15.00	0.00	0.20				
Expectation	6.43	17.14	11.42				

H=Heterozygosity; χ^2^=Chi-square;

* = p<0.05

The test of significance showed that the fixed effect of genotype together with group effect of farm and linear and quadratic coefficients of age were statistically significant (p<0.05) in BW, CW, HW, and HG. On the other hand, the fixed effect of genotype, type of birth, and linear and quadratic coefficients of age were statistically significant (p<0.05) in HH (Table-3). Furthermore, statistical analysis of the association of genotype with BW and BM showed that animals of genotype GG were significantly heavier and larger (p<0.05) than animals of genotype CC: The superiority of BW7 was 1.89 kg (GG 11.05 kg vs. CC 9.16 kg) and the superiority of BW8 was 1.86 kg (GG 11.76 kg vs. CC 9.9 kg). Nonetheless, no significant differences were observed in BW1-BW6, although animals of genotype GG tended to be heavier. In terms of BM, significant differences were observed at the following ages: CW3, CW7, CW8, HH4, HH7, HW7, HW8, HG2, HG4, HG5, HG7, and HG8 (Table-4).

**Table-3:** Significance analysis of factors affecting body weights and body measurements.

Traits	Effect	Degree of freedom	f-value	p-value
BW	Genotype	1	4.61*	0.0328
	Group of farm	3	4.42*	0.0048
	Age	1	235.2*	<0.0001
	Age*age	1	19.33*	<0.0001
CW	Genotype	1	8.26*	0.0044
	Group of farm	3	5.86*	0.0007
	Age	1	39.44*	<0.0001
	Age*age	1	10.58*	0.0013
HH	Genotype	1	5.46*	0.0202
	Type of birth	2	3.14*	0.0346
	Age	1	275.95*	<0.0001
	Age*age	1	62.75*	<0.0001
HW	Genotype	1	4.38*	0.0397
	Group of farm	3	2.21*	0.0875
	Age	1	60.78*	<0.0001
	Age*age	1	12.50*	0.0005
HG	Genotype	1	5.37*	0.0214
	Group of farm	3	4.54*	0.0041
	Age	1	362.96*	<0.0001
	Age*age	1	74.62*	<0.0001

BW=Body weight, CW=Chest width, HH=Hip height, HW=Hip width, HG=Heart girth

**Table-4:** Estimated genotypic effect on body weights and body measurements by linear mixed model analysis.

Traits and measurement at eight periods	Genotypes	

	GG	CC
Body weight		
BW1	4.12±0.21	3.78±0.19
BW2	5.32±0.27	4.96±0.25
BW3	6.62±0.35	5.98±0.33
BW4	8.02±0.42	7.02±0.38
BW5	9.19±0.47	7.80±0.43
BW6	10.10±0.52	8.57±0.48
BW7	11.05±0.56^a^	9.16±0.52^b^
BW8	11.76±0.60^a^	9.90±0.55^b^
Chest width		
CW1	8.60±0.30	8.61±0.28
CW2	10.56±0.64	9.63±0.58
CW3	10.40±0.25^a^	9.52±0.23^b^
CW4	10.78±0.26	10.12±0.23
CW5	11.19±0.27	10.38±0.24
CW6	11.40±0.27	10.59±0.24
CW7	11.87±0.27^a^	10.58±0.25^b^
CW8	12.07±0.31^a^	10.80±0.29^b^
Hip height		
HH1	37.37±1.39	35.75±0.88
HH2	40.33±1.15	39.09±0.72
HH3	43.19±1.30	42.46±0.82
HH4	47.53±1.14^a^	44.36±0.71^b^
HH5	47.85±1.23	46.15±0.77
HH6	49.33±1.22	47.24±0.77
HH7	51.26±1.33^a^	48.01±0.84^b^
HH8	52.59±1.41	50.04±0.89
Hip width		
HW1	8.13±0.26	7.76±0.24
HW2	9.05±0.25	8.01±0.23
HW3	9.33±0.25	8.65±0.22
HW4	9.60±0.52^a^	9.85±0.48
HW5	10.07±0.30	9.98±0.27
HW6	10.31±0.27	9.96±0.25
HW7	11.06±0.28^a^	9.89±0.26^b^
HW8	11.55±0.26^a^	10.11±0.24^b^
Heart girth		
HG1	32.54±0.84	31.96±0.79
HG2	36.75±0.78^a^	34.07±0.71^b^
HG3	38.75±0.79	37.23±0.73
HG4	42.03±0.80^a^	38.94±0.73^b^
HG5	44.84±0.88^a^	40.57±0.80^b^
HG6	44.86±1.01	43.15±0.93
HG7	46.37±0.82^a^	43.48±0.76^b^
HG8	48.21±0.88^a^	44.29±0.80^b^

In the same row, values with different superscripts are significantly different (p<0.05)

In this study, NLMM was used to compare the growth models of the two genotypes in Kejobong goats. Estimated parameters of non-linear growth model and the result of fitness statistics for BW, HG, CW, HH, and HW, in Table-5, showed that the von Bertalanffy model was the best for describing BW, the Brody model for CW and HH, the Gompertz and Logistic models for HG, and the von Bertalanffy together with the Gompertz model for HW.

**Table-5:** Estimated parameters of growth and goodness of fit for four different growth model for body weight and body measurements.

Variable	Parameter	Model			

		Brody	Von Bertalanffy	Logistic	Gompertz
Body weight	A	26.6165±0.8838	24.5848±0.4119	23.3771±0.2494	24.119±0.3392
	B	0.8293±0.01063	0.394±0.006254	2.5904±0.07293	1.4256±0.02609
	C	0.007131±0.00103	0.01416±0.00111	0.02815±0.001329	0.01766±0.001158
	σ^2^_u_	8.0152±2.3596	5.1533±1.3331	3.7526±0.9346	4.5865±1.1652
	σ^2^_e_	0.3224±0.02913	0.3213±0.02903	0.3263±0.02948	0.3218±0.02907
	GG	−3.6102±0.8251	−4.9019±0.5525	−5.671±0.4451	−5.1983±0.5074
	CC	−6.3733±0.6891	−7.1133±0.5099	−7.552±0.4252	−7.2827±0.4759
	−2 Log-likelihood	607.7	606.9	610.7	607.2
	AIC	629.7	628.9	632.7	629.2
	BIC	646.8	646.0	6.49.8	646.3
Heart girth	A	54.4743±0.7187	53.2477±0.6747	53.3279±0.4993	52.5766±0.5957
	B	0.4103±0.01058	0.1565±0.004488	0.6127±0.01979	0.5006±0.01461
	C	0.01420±0.001606	0.01654±0.001683	0.02211±0.001747	0.01798±0.001687
	σ^2^_u_	7.7466±2.0471	10.8093±4.328	7.0365±1.8202	7.4281±1.9557
	σ^2^_e_	2.9037±0.2623	2.8952±0.2609	2.9038±0.2623	2.9024±0.2622
	GG	−0.1247±0.7312	0.3109±0.8102	−0.6902±0.6498	0.6090±0.6901
	CC	−3.001±0.6906	−2.6632±0.7726	−3.5819±0.6211	−2.6324±0.6511
	−2 Log-likelihood	1186.7	1188.6	1186.6	1186.5
	AIC	1208.7	1210.6	1208.6	1208.5
	BIC	1225.8	1227.7	1225.7	1225.7
Chest width	A	13.2044±0.2047	13.1842±0.1938	14.0087±0.1767	13.6037±0.1890
	B	0.3014±0.02035	0.1105±0.008294	0.4026±0.03666	0.3477±0.02746
	C	0.02339±0.006807	0.02526±0.0066994	0.02897±0.007371	0.02619±0.007088
	σ^2^_u_	0.3148±0.1220	0.3127±0.1212	0.3093±0.1198	0.3117±0.1208
	σ^2^_e_	1.2031±0.1087	1.2046±0.1088	1.2076±0.1091	1.2054±0.1089
	GG	0.2807±0.1912	0.2693±0.1871	−0.3206±0.1809	−0.02153±0.1853
	CC	−0.6763±0.1736	−0.6851±0.1709	−1.2707±0.166	−0.9747±0.1697
	−2 Log-likelihood	880.9	881.2	881.9	881.4
	AIC	902.9	903.2	903.9	903.4
	BIC	920.0	920.4	921.0	920.5
Hip height	A	60.9893±0.6814	60.7061±0.6247	58.01±0.551	58.3122±0.6023
	B	0.3611±0.009661	0.1349±0.003943	0.5129±0.01791	0.4291±0.01311
	C	0.01687±0.002045	0.01922±0.002103	0.02395±0.002234	0.02040±0.002134
	σ^2^_u_	7.9053±2.0794	7.7528±2.0352	7.5263±1.9715	7.6877±2.0166
	σ^2^_e_	3.9857±0.3601	3.9963±0.3610	4.0208±0.3633	4.0021±0.3616
	GG	−1.9251±0.7249	−2.0781±0.7042	−0.9427±0.6775	−0.7799±0.6961
	CC	−4.6856±0.5687	−4.8157±0.5513	−3.6473±0.5289	−3.5079
	−2 Log-likelihood	1269.5	1270.2	1271.7	1270.5
	AIC	1289.5	1290.2	1291.7	1290.5
	BIC	1305.1	1305.7	1307.2	1306.1
Hip width	A	12.7384±0.3135	14.7988±0.2714	12.5354±0.2168	13.4782±0.2548
	B	0.3621±0.02307	0.1349±0.009079	0.51±0.03891	0.4284±0.0297
	C	0.01499±0.004015	0.01737±0.00421	0.02213±0.00436	0.01855±0.004177
	σ^2^_u_	0.5232±0.1647	0.5104±0.159	0.4918±0.1515	0.505±0.1568
	σ^2^_e_	0.7385±0.06676	0.7385±0.06675	0.7385±0.06676	0.7385±0.06675
	GG	0.4472±0.2464	−1.0273±0.2289	0.3342±0.2075	−0.1896±0.2222
	CC	−0.3087±0.2203	−1.7739±0.2073	−0.3988±0.1915	−0.9322±0.2023
	−2 Log-likelihood	765.3	765.3	765.3	765.3
	AIC	787.3	787.3	787.3	787.3
	BIC	804.5	804.4	804.5	804.4

AIC=Akaike information criterion, BIC=Bayesian information criterion

## Discussion

In this study, the lack of HWE beside high heterozygosity showed that the population was under selection pressure. These goats are not mated randomly with respect to locus and experience migration, natural selection, mutation, or genetic drift [21]. In this study, the absence of GC heterozygous genotypes is suspected because animals of heterozygous genotypes have smaller BW than those of animals of homozygous genotype. Hence, breeders generally tend to choose or maintain animals that have larger BW and culling the animal that has a smaller BW. Animals of genotype GG showed higher BW (p<0.05) than those of genotype CC, however, that became evident close to weaning age (BW7 and BW8); BM also tended to demonstrate significant differences only in the last two measurements (96-111 days old and 112-127 days old). These results were consistent with those of a previous study [22], where significant differences among genotypes in *IGF1* show at 3-12 months old for BW of buffalos, whereas at 0-3 months old, no significant differences appeared. In this study, the high weaning weight was assumed to produce a high rate of post-weaning weight gain, which is in accordance with the finding that *IGF1* concentration gradually increases toward weaning age and decreases after maturity [23].

In this study, SNP found at intron 4 had an effect on BW and BM at late ages. Although the intron is a genome in the non-coding region, it plays an important role during transcription, such as transcription rate, initiation, termination, regulation, and alternative splicing [24]. Therefore, SNP at the intron may affect the structure and function of the protein, gene expression levels and animal psychological metabolism, all of which influence animal growth. In this study, SNP was located as described in Nanjiang Huang goats [9] and in Markhoz goats [25]. Another study has emphasized the role of SNPs located in the intron region in nine Indian goat breeds, disclosing two of eight novel SNPs within the intron of the *IGF1* gene that has a significant association with BW at different ages [26]. In this study, therefore, SNP at intron position (g5752G→C) was considered one of the genetic markers for the selection of BW and BM in Kejobong goats.

Parameters AIC, BIC, and −2 log-likelihood for BW implied that the lowest value in the von Bertalanffy model was the best fitted to the growth model (Table-5). This differed from the Gompertz and Brody models which clearly explain the growth of Beetal goats [8]. There were diverse results in choosing the best model that can be attributed to the variations in mathematical formulae of equations, the number of records, and the amount of data observed and record collecting intervals [27,28]. In this study, the Brody model showed the lowest values of AIC, BIC, and −2 log-likelihood in CW and HH, compared with the von Bertalanffy, Logistic, and Gompertz models. The foregoing results suggest that the Brody model is the best for estimating the CW and HH. The Gompertz model which was the best for the HG showed the lowest values of AIC, BIC, and −2 log-likelihood and was very similar to those of the Logistic model. Therefore, these models were considered as the best for describing HG in Kejobong goats. Similarly, the von Bertalanffy and Gompertz models were considered as the best for describing HW.

For describing BW, the von Bertalanffy model was the best for estimating mature BW (A) (24.58 kg), integration constant (B) (0.394), and growth rate constant (C) (0.01416), while the highest and the lowest estimated parameter A was observed under the Brody (26.61 kg) and Logistic (23.37 kg) models, indicating that Kejobong goats have a lower mature BW than Markhoz goats (30.50 kg) [29]. The best-estimated parameter A for CW and HH was 13.20 cm and 60.98 cm, respectively, while for HG it was 52.57 cm (Gompertz model) and 53.32 cm (Logistic model) and for HW it was 14.79 cm (von Bertalanffy model) and 13.47 cm (Gompertz model). The estimated value of the parameter A does not imply the highest weight attained by individuals; it only indicates the average weight of mature individuals [30]. In this study, the estimated parameter B ranged between 0.39 and 2.59 for BW, and between 0.11 and 0.61 for BM. Parameter B is a scale parameter that has no biological interpretation [31]. In the present study, parameter C showed the growth rate reaching mature BW; thus, the largest parameter C was less likely to reach a great mature BW; in other words, animals that were heavy at mature age tended to go through a slower growth rate. This result is consistent with the previous reports that large weights at maturity are associated with small growth rates [29] and that the weight at maturity and the growth rate have a highly negative genetic correlation [7]. In this study, the estimated residual variance of BW was equivalent among the models. Residual variance described the gap between the predicted value and observed value. The estimated animal variance of the BW under the Brody, von Bertalanffy, Logistic, and Gompertz models was 8.01, 5.15, 3.75, and 4.58, respectively. Animal variance indicates variability among individual animals: The higher the variance, the greater the difference among them.

Animals of genotype GG demonstrated greater BW and larger BM than those animals of genotype CC; however, significant differences were observed at only later stages of animal growth, which may be attributable to the limited number of observations, suggesting that significant differences prevailed from early stages of growth to maturity. Growth analysis under NLMM can reduce the influence of potential biases despite selective sampling and can supply supplemental parameters that characterize variation between individual animals [32]. Therefore, these considerations provide proper and accurate estimates of parameters to describe the growth performance of Kejobong goats.

## Conclusion

SNP at intron 4 (g5752G→C) in the *IGF1* gene is associated with growth traits and can be used as MAS for the improvement of these traits. Greater BW and larger BM were demonstrated by animals of genotype GG when they approach weaning age. The von Bertalanffy model (y = 24.58 (1–0.39 Exp^–0.014age^)^3^) was the best for describing BW, the Brody model for CW (y = 13.20 (1–0.30 Exp^–0.023age^)) and HH (y = 60.98 (1–0.36 Exp^–0.016age^), the Gompertz (y = 52.57 Exp (–0.50 Exp^–0.017age^)) and Logistic (y = 53.32/(1+0.61 Exp^–0.02age^)) models for HG, and the von Bertalanffy (y = 14.79 (1–0.13 Exp^–0.017age^)^3^) together with the Gompertz (y = 13.47 Exp (–0.42 Exp^–0.018age^) model for HW. Further study is needed to validate our results with a larger number of animals and recorded sample observations, especially at later stages of growth.

## Authors’ Contributions

DAL: Designed the study, collected data, interpreted data analysis, drafted the manuscript; TO: Interpreted data analysis; SS: Interpreted data analysis; EP: Supervised the work; AS: Critical data analysis; EK: Supervised the work, critical construction of the manuscript. All authors read and approved the final manuscript.
